# Monitoring the Diversity and Metabolic Shift of Gut Microbes during Green Tea Feeding in an In Vitro Human Colonic Model

**DOI:** 10.3390/molecules25215101

**Published:** 2020-11-03

**Authors:** Mengyang Xu, Kundi Yang, Jiangjiang Zhu

**Affiliations:** 1 Department of Chemistry and Biochemistry, Miami University, Oxford, OH 45056, USA; xum@miamioh.edu (M.X.); yangk@miamioh.edu (K.Y.); 2 Human Nutrition Program, Department of Human Sciences, The Ohio State University, Columbus, OH 43210, USA; 3 James Comprehensive Cancer Center, The Ohio State University, Columbus, OH 43210, USA

**Keywords:** gut microbiota, green tea, polyphenol, metabolites, human colonic model

## Abstract

The human gut microbiome plays an important role in human health, and many factors such as environment, host genetics, age, and diet have been found to influence the microbial composition. Tea, as one of the widely consumed beverages, has been known for centuries to have antioxidant, anti-inflammatory, and anticancer effects. To investigate the impact of green tea polyphenol on the diversity and metabolic functions of human gut microbes, we applied an in vitro human colonic model (HCM) in this study to mimic a short-term green tea ingestion event and investigate its related changes to gut microbial composition and their metabolic functions. The pH, temperature, anaerobic environment, feeding nutrient, and time point in each compartment of the HCM were tightly controlled to simulate the intestinal system, and pooled human fecal samples of two healthy volunteers were used for the colon microbiota inoculation within the colonic model. By adding green tea extract (GTE) to the growth medium, the detailed impacts of GTE polyphenol on gut microbial population/diversity, gut microbial metabolites, metabolic pathways, and their associations were investigated via 16 S ribosomal DNA sequencing and liquid chromatography coupled tandem mass spectrometry (LC-MS/MS) analyses. Our data indicated that the treatment of green tea extract applied to gut microbiota can induce a significant decrease in the abundance of *Firmicutes* and a slight decrease in the abundance of *Bacteroidetes*, and these changes result in a decreased *Firmicutes/Bacteroidetes* ratio, which can be an effective indicator for successful GTE intervention, which may generate beneficial health effect to human. Meanwhile, the relative abundances of many detected bacteria genera among three HCM vessels changed through the GTE intervention. The overall effects of GTE on gut microbial beta-diversity were observed by multivariate statistical analyses, and the differences in metabolic profiles from different GTE treatment stages were detected. Moreover, we identified several associations between microbial population and microbial metabolites, which may assist us in establishing new hypotheses for future related studies. In summary, our study suggested that the microbial compositional changes induced by GTE also changed their metabolic functions, and consequentially, may change the host metabolism and impact human health.

## 1. Introduction

The human-associated microbiota are a complex community that contains over 100 trillion microorganisms, including bacteria, fungi, viruses, and archaea, colonizing all of the surfaces of the human body that are exposed to the environment, with the majority residing in the digestive tract which we call the gut microbiota [1,2]. Over the past decade, the gut microbiota have re-emerged as an important research subject. These microbes can influence host metabolic, physiology, nutrition, and immune pathways which play an important role in human health and disease [3,4]. The gut microbiota are formed from infancy and continue to mature along with the host [3]. Many factors have been found to influence the microbial composition, such as environment, host genetics, age, and diet [5]. Among these, dietary changes have a very significant influence on the diversity and composition of gut microbiota [6]. For instance, a study has shown that microbiota can be changed within a day by feeding a high-fat, high sugar “Western” diet to mice [7]. Studies also showed that the manipulation of gut microbiota by using prebiotics mixtures can improve hepatic steatosis, inflammation, and insulin sensitivity in rodents [8,9]. These studies indicated that the gut microbiota community plays an important role in nutritional intake and biotransformation, the regulation of lipid metabolism, as well as in inflammatory pathways [10].

Tea, as one of the most widely consumed beverages, has been known for centuries to have antioxidant, anti-inflammatory, and anticancer effects [11,12,13]. Black tea, green tea and Oolong tea are major types of tea that are generally consumed by millions of people every year. The production methods and chemical composition can be used to differentiate them, and green tea consists of large amounts of flavan-3-ols known as catechins, epigallocatechin-3-gallate (EGCG) and other phenolic compounds [14,15,16]. These phenolic compounds extracted from tea make major contributions to tea’s beneficial effects; almost 95% of one’s polyphenols daily intake (ranging from less than 100 mg to over 2 g) is expected to reach the colon and is fermented by gut microflora [17]. Green tea phenolic compounds showed great antimicrobial effects and an ability to alter gut microbiota in favor of the polyphenol-metabolizing components of the microbiota [18]. Studies also showed that the antimicrobial ability of green tea phenolic compounds can bypass beneficial bacterial, such as lactic acid bacteria, but selectively target the growth of pathogenic bacteria [19,20,21]. While some bio-transformations have been suggested to enhance the bio-availability of polyphenols, such as catabolic breakdown, methylation, deglycosylation, etc., via the microbial community [22], a better effort is now needed to systematically identify the variety of microbial metabolites produced, the commensal microbes responsible for their production, and their potentially beneficial biological activities.

As the metabolic transformation processes during gastrointestinal tract digestion are complex, the small intestine, colon and liver have all been reported to participate in the absorption and biotransformation of polyphenols [23]. However, studies have also suggested that while dietary polyphenols can be absorbed in the small intestine, the absorption rate is relatively low (10–20%) [23], and this leaves a rather large amount of polyphenols to be further biotransformed by the huge population of gut microbes in the colon [24,25,26]. Furthermore, in order to investigate the interactions between the tea polyphenols and the gut microbiota, the colon is naturally the best option, as it is the hub of a large gut microbial population compared to the small intestine. Both in vivo and in vitro colon models have been used in the past to investigate the gut microbial biotransformation of dietary components. Due to various restraints and ethical considerations, human studies of gut microbial metabolites tend to be correlative and inconclusive [27]. Animal models, particularly of germ-free and gnotobiotic animals, have led to insightful discoveries [28,29], but major drawbacks, such as high costs and low throughputs, still exist [30]. Alternatively, the gut microbe metabolome can be examined in in vitro colonic models with carefully designed experiments [31,32]. The colonic models can be “humanized” by colonizing them with human intestinal microbes, providing an exciting tool for examining the function of a specific human microbiota and testing how the microbiota interact with specific perturbations and other environmental factors that influence gut microbial metabolism. In vitro colonic models have become more important and popular in the human microbiota research in recent years [31,33,34,35,36]. The strict control of this complex system provides a gut microbial ecosystem that makes it easier to monitor metabolic products, and less complicated to interpret the biological origins of these metabolites. Multiple-stage continuous colonic culture fermentation models are so far the most effective systems. With these models, different vessels can be separately controlled to mimic different sections of the colon, thereby allowing manipulation of the gut microbe, helping identify the specific molecular mechanisms by which the gut microbiota and their metabolism are affected [34,37,38].

In this study, we utilized an in vitro human colonic model (HCM) system that involves the control of pH, temperature, anaerobic environment, feeding nutrient, and feeding time points to simulate the large intestinal system for nutritional studies [33,39]. By seeding human feces-derived microbial communities from healthy human hosts into the HCM system, and strictly controlling the environmental parameters, the established microbial community can be studied for a variety of perturbations. By adjusting the “diet” of the system to the addition of green tea-extracted phenolic compounds, we are able to investigate the detailed impact of green tea extract on gut microbial population/diversity, gut microbial metabolites, and their associations. It also allows us to discover polyphenol metabolites from gut microbial fermentation, and enables the detailed study of the metabolic functions of the human gut microbiota.

## 2. Materials and Methods

### 2.1. Chemicals

Myricetin, 3,4-dihydroxy phenyl ethanol, homovanillic acid sulfate, equol, genistein, phloretin, kaempferol, enterolactone, hesperetin, isorhamnetin, protocatechuic acid, trans-resveratrol, roseoflavin, pyrogallol, isoferulic acid, urolithin A, phloroglucinol, (R)-γ-valerolactone and freight were purchased from Cayman company (Ann Arbor, MI, USA). Other polyphenol standards were purified in-house through chromatographic methods with mass spectrometry/NMR confirmation. Authentic standards corresponding to the measured metabolites were purchased from Sigma-Aldrich (Saint Louis, MO, USA) or IROA Technologies (Boston, MA, USA). The stable isotope-labeled amino acid mix (20 AA U-13C, 97–99%; U-15N, 97–99%) was purchased from Cambridge Isotope Laboratories (Tewksbury, MA, USA). HPLC-MS-grade acetonitrile, ammonium acetate, and acetic acid were all purchased from Fisher Scientific (Pittsburgh, PA, USA).

### 2.2. Human Colon Microbiota

Following an extraction procedure published in a previous study, the pooled human fecal samples of two healthy volunteers were used for culturing colon microbiota [29]. Approval was obtained from the Institute Review Board (IRB) committee of Miami University before the study (project reference number 02223e), and informed consent was obtained from the healthy fecal donors. The fecal content was vortexed and suspended in pre-reduced PBS with 0.1% cysteine. The diluted suspension was plated on a Gifu Anaerobic (GAM) Agar plate (HiMedia Laboratories LLC, West Chester, PA, USA) for 48 h at 37 °C in an anaerobic environment inside a type A vinyl anaerobic chamber (COY lab, Grass Lake, MI, USA) that was operated according to the manufacturer’s recommended protocols. The GAM medium contained a peptic digest of animal tissue, protease peptone, digested serum, yeast extract and other ingredients to provide sufficient nutritional components for gut microbial growth. It has been reportedly used for growing 32 dominant species of human gut microbes successfully in a previous report [39]. A sterile wire loop was used to select colonies from the agar plate. Colonies from GAM plates were picked and incubated in 5 mL GAM broth for 48 h under the same condition. Bacteria frozen stocks were then prepared by mixing the bacteria culture and 20% glycerol, which was then frozen in a −80 °C freezer until use.

### 2.3. In Vitro Human Colonic Model (HCM)

**Figure 1:**
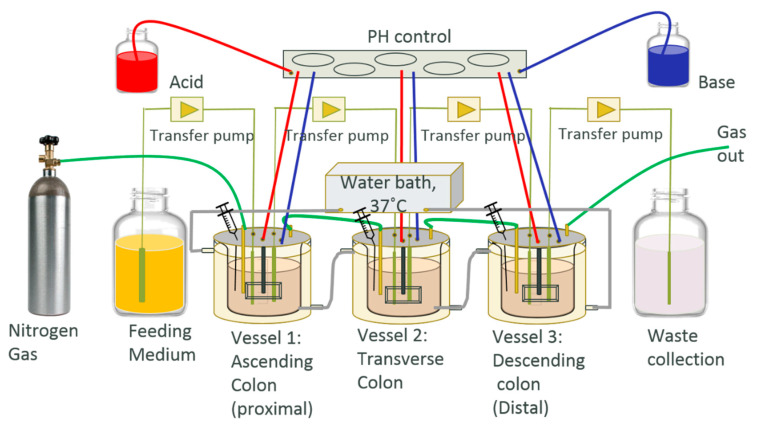
In vitro human colonic model (HCM) contains three vessels. vessel 1 (V1) has a 1 L operating volume at pH 5.6, V2 has a 1.6 L operating volume at pH 6.2, and V3 has a 1.2 L operating volume at pH 6.8, which mimic the environmental condition of the ascending colon, transverse colon and descending colon, respectively.

The HCM system in this study was designed based on the three-stage continuous culture system in a previous study [33]. It contains three vessels; vessel 1 (V1) has a 1 L operating volume at pH 5.6, V2 has a 1.6 L operating volume at pH 6.2, and V3 has a 1.2 L operating volume at pH 6.8, which mimic the environmental condition of the ascending colon, transverse colon and descending colon, respectively (Figure 1). The culture system was inoculated with fresh feeding medium three times a day at the volume of 100 mL each time, to mimic the feeding of breakfast, lunch and dinner time. There is a 1 h delay time between the feeding medium being transferred from V1 to V2, and V2 to V3, to allow sufficient time for the microbial metabolism of nutrients in each vessel. The feeding medium initially contained GAM for the equilibrium period and 0.67 mg/mL green tea extract was added into the GAM medium for the treatment period that was recalculated based on the references [40]. An offline stomach process included a one-hour incubation of medium at pH 2 in 37 °C, followed by a two-hour incubation with the addition of digestive enzyme at pH 5.5 in 37 °C for digestion, and feeding content was applied before the medium was added into the system [41]. The vessels were water-jackets with warm water at 37 °C, supplied by a program-controlled circulating water bath. N_2_ gas was used to maintain the anaerobic atmosphere by constantly bubbling into the vessels, pH solutions, and feeding medium at 0.5 L/min. Gastrointestinal motility was mimicked in each vessel by using an automatic controlled-speed stir rod, and the pH of each vessel was adjusted automatically by pumping acidic and basic solutions into each vessel via our in-house LabView program. Samples of each vessel were collected through sampling ports once a day. The bacteria cell number was obtained by applying a drop plate method based on a published study [42].

### 2.4. Green Tea Extraction

Green tea was purchased from a local tea retailer. Green tea extract (GTE) was prepared as in the method of Zhao and Shah, with modification [43]. Briefly, crude dry tea leaves were infused in boiling deionized (DI) water in a ratio of 1:20 (*w*/*w*) for 20 min. A Buchner funnel with filter paper was used for filtering the crude green tea extract to exclude tea leaves. Small particles in GTE were removed by filtering through a Nalgene™ Rapid-Flow™ sterile disposable bottle top filter of 0.2 µm pore size. The extract was lyophilized and the freeze-dried powder was stored at −80 °C before use.

### 2.5. 16 S rRNA Gene Library Preparation and Analysis

In this study, 16 S ribosomal RNA gene sequencing following an established protocol [44,45] was used to do the metagenome analysis of culture samples. The V4 region of the 16 s rRNA gene (primers 515F and 806R) was amplified using the GoTaq^®^ Hot Start Colorless Master Mix (Promega, Madison, WI, USA). Each DNA sample was amplified using a reverse primer tagged individually with a unique 12-base Golay barcode. The PCR amplification program was as follows: 1 cycle of 94 °C for 3 min; 35 cycles of 94 °C for 45 s, 50 °C for 60 s and 72 °C for 90 s; 1 cycle of 72 °C for 10 min; 1 cycle of 4 °C for 5 min. Amplicons were purified by the SequalPrep Normalization Plate kit (Thermo Fisher, Waltham, MA, USA). Purified products were quantified by KAPA Library Quantification Kit Illumina Platforms (Kapa Biosystems, Wilmington, MA, USA). Pooled libraries were sequenced on the Illumina MiSeq platform using a read length up to 2 × 250 bp.

### 2.6. Metabolites Extraction

The metabolite extraction of intracellular cells was prepared using a cold methanol (−20 °C) method following the sampling protocol from our previous work [46]. Briefly, 2 mL of bacteria culture was followed by two rapid centrifugations and a 0.5 mL phosphate buffer saline (PBS) wash. A quantity of 250 μL of methanol that contained a mix of ^13^C^15^N labeled internal standards was added to the cell pellet in the tube, and the samples were vortexed vigorously for 2 min. One hundred fifty microliters of supernatant was collected and dried by using a Eppendorf Speedvac system (Enfield, CT, USA) after incubation at −20 °C and centrifugation. Samples were then reconstituted by a 1:1 mixture of acetonitrile and ultrapure water, and loaded into liquid chromatography vials for analysis.

### 2.7. Polyphenol Extraction

The polyphenol extraction method was modified from previous work [47]. Sulfatase (from *Helix pomatia*) and β-glucuronidase (from *Escherichia coli*) were purchased from Sigma-Aldrich. Other reagents and HPLC-grade solvents were purchased from Thermo Fisher Scientific. Briefly, 2 mL of bacterial culture from HCM was collected and centrifuged at 19,722× *g* for 5 min to remove the supernatant. Quantities of 1 mL of 10% Ascorbic acid and 0.1% EDTA in 0.4 M NaH_2_PO_4_ were used to reconstitute the pellet, and 20 μL of enzyme mix (240 units β-glucuronidase and 10 units sulfatase) was added, then it was warmed at 37 °C with aerobic shaking at 180 rpm for 45 min. The reaction was stopped by adding 200 μL methanol followed by adding 500 μL chloroform, vortexing for 4 min, and centrifuging at 14,000 rpm for 5 min to remove lipid compounds. Ethyl acetate was used to extract polyphenol from the aqueous phase and the extraction was vacuum dried at 30 °C for 1 h. The dried extracts were reconstituted with 150 μL 15% ACN-H2O, and after being centrifuged at 14,000 rpm for 5 min, 100 μL of supernatant was collected in LC vials for analysis.

### 2.8. Targeted HPLC-MS/MS Metabolic Profiling

The targeted metabolite compound detection approach applied in this study was similar to our previous work [48]. Briefly, a Thermo Scientific TSQ Quantiva triple quadrupole mass spectrometer (Waltham, MA, USA) equipped with an electrospray ionization source, applied for both positive and negative mode compound detection, was coupled with a Thermo Scientific Ultimate 3000 high-performance liquid chromatograph (HPLC, Waltham, MA, USA), equipped with an amide hydrophilic interaction chromatograph (HILIC) column with dimensions of 2.1 × 150 mm and a particle size of 2.5 μm (Waters Corporation, Milford, MA, USA), for metabolite detection, and an Xterra RP-C18 column (Waters Corporation, Milford, MA, USA) with dimensions of 3.9 × 100 mm and a particle size of 3.5 μm was used for polyphenol detection. For metabolites detection, the extracted bacterial intracellular reconstituted samples were injected into the column for gradient elution separation at 0.300 mL/min using solvents A (5 mM ammonium acetate in 90% water/10% acetonitrile/0.2% acetic acid *v*/*v*/*v*) and B (5 mM ammonium acetate in 90% acetonitrile/10% water/0.2% acetic acid *v*/*v*/*v*). The autosampler temperature was kept at 4 °C, the column compartment was set at 40 °C, and the separation time for each sample was 20 min. The retention time and selected reaction monitoring (SRM) transitions of targeted metabolites and polyphenol (or compound pairs) were established by running pure standards and collecting the tandem mass spectra (MS/MS); therefore, the orthogonal information on the retention time and two pairs of SRM transitions can be used to confidently detect and identify targeted compounds [49,50]. A stable isotope-labeled amino acid mix was also used for quality control purposes during MS runs. This method has been checked and validated monthly to ensure its performance, and a list of the detected intracellular metabolites is provided in Supplementary Table S1. When biological samples were run, pooled quality control samples were also tested in between every 10 samples to monitor the instrument’s stability.

### 2.9. Targeted HPLC-MS/MS Phenolic Compounds Profiling

The same HPLC-mass spectrometry system mentioned above was used in the targeted phenolic compound detection, and this time an Xterra RP-C18 column (Waters Corporation, Milford, MA, USA) with the dimensions of 3.9 × 100 mm and a particle size of 3.5 μm was used for optimal separation. Extracted bacterial samples were injected into the column for gradient elution separation at 0.900 mL/min using solvents A (89.9% water/10% acetonitrile/0.1% formic acid, *v*/*v*/*v*) and B (69.9% water/30% acetonitrile/0.1% formic acid, *v*/*v*/*v*). The autosampler temperature was kept at 4 °C, the column compartment was set at 30 °C, and the separation time for each sample was 11 min. The retention time and selected reaction monitoring (SRM) transitions of targeted metabolites and polyphenols (or compound pairs) were established by running pure standards and collecting the MS/MS data, and therefore, the orthogonal information on the retention time and two pairs of SRM transitions can be used to confidently detect and identify phenolic compounds. When biological samples were run, pooled polyphenol standards as quality controls were also tested in between every 10 samples to monitor the instrument stability.

### 2.10. Statistical and Bioinformatics Analysis

The newest version Quan Browser module of Xcalibur (Thermo Fisher Scientific, Waltham, MA, USA) was used to process the raw metabolite data generated from HPLC–MS/MS analysis. Seventy-seven metabolites (Supplementary Table S2) related to frequently investigated pathways such as TCA Cycle and glycolysis had detectable signals in more than 75% of the samples. JMP Pro12 (SAS Institute, Cary, NC, USA) was used for statistical analysis following the recommended protocols [51]. Metabolic profiles were analyzed using an online tool MetaboAnalyst 3.0 (http://www.metaboanalyst.ca/) [52]. Principle component analysis (PCA) was applied for the metabolic profile comparison of control samples from the equilibrium period to nanoparticle-treated samples. Bar charts were generated by excel using relative abundance. The heatmap of polyphenol concentration was generated in R Bioconductor using the heatmap.2 function of the gplots package (http://cran.rproject.org/web/packages/gplots/index.html). Microbial composition analysis based on 16 S rRNA data was performed using the software QIAGEN CLC Genomics Workbench (v11.0.1, Germantown, MD, USA). First, read pairs were imported and combined in silico. For OTU clustering, samples were filtered based on the number of trimmed reads (0.03 quality limit). Samples with read numbers above 10,000 and above 50% from median were aligned against the SILVA database version 128 with 97% similarity. A sample description with group information was imported and associated with reads to generate the abundance table. A beta diversity analysis tool performed principal coordinate analysis (PCoA) on the distance matrices based on aligned OTUs and an appropriate phylogenetic tree. JMP also performed Spearman correlation analysis between microbial genus abundances and metabolites in rat fecal samples. Phylogenetic investigation of communities by reconstruction of unobserved states (PICRUST, Galaxy V1.1.1) was used to predict microbial function [53]. Briefly, the Illumina sequencing data were processed using quantitative insights into microbial ecology (QIIME, V 1.9.1) [54], and the sequencing reads were assigned to species equivalent operational taxonomic units (OTUs) (at 97% sequence similarity) using a closed-reference OTU-picking strategy with GreenGenes (gg_13_08) as our reference [55]. The resulting OTU abundance table was normalized to the 16 S rRNA gene copy number from known bacterial genomes in integrated microbial genomes. The Kyoto Encyclopedia of Genes and Genomes (KEGG) database was aligned to predict gene function. Statistical Analysis of Metagenomic Profiles (STAMP, V2.1.3) was used to analyze the functional data with removed unclassified reads. The differences between groups were determined using Welch’s *t*-test with a *p*-value filter of *p* ≤ 0.05. Storey’s FDR approach was applied to all features. The features with *q*-values above 0.005 and the features with small effect sizes were removed by filtering with the difference between proportions below 0.2.

## 3. Results and Discussion

### 3.1. Design of Human Colonic Model (HCM) System

Based on previously published studies [33,39], we built a human colonic model (HCM) system to specifically study the microbial ecosystems. As shown in Figure 1, the system has three fermentation vessels that mimic the environmental condition of the ascending colon, transverse colon, and descending colon, respectively. The HCM was also equipped with computer-programmed automatic controls for pH, temperature, anaerobic environment and feeding process to be an ideal system for us to study the microbial population, diversity and metabolic changes after any desired nutritional/drug intervention without any complicating factors from the host. Moreover, the system maintains no contamination before the inoculation thanks to different types of sterilization (autoclave sterilization, alcohol sterilization, and ultraviolet radiation). Each vessel was inoculated by gut microbes that were derived from healthy donors’ feces. The microbial community was allowed to establish equilibrium (until the bacteria cell number became stable) before the experiment started. According to the OTU summary, more than 75% of the bacteria phylum and 62% of the bacteria genera from the original human fecal sample were recovered in the HCM. Once the bacteria cell number became stable (pretreatment period), the experiment started and the samples were collected, and the data were compared in the treatment period and post-treatment period, to investigate the green tea polyphenol impact on gut microbes. The GAM medium containing 0.67 mg/mL green tea extract was used to feed microbes during the treatment period, while regular GAM medium was used during the pretreatment period and post-treatment period, respectively [56].

Most studies of human-associated microbiota used human and animal models [5]. However, the limitations of using these models, including possible species differences (when comparing the animal model to human host gut microbes), high costs, unrealistic dosages and exposure durations, and many health effects, are multifactorial [57]. Additionally, human and animal models are always limited by end-point measurements for dynamic monitoring [58]. Compared to in vivo human and animal models, the HCM system can strictly control the environment parameters and can be sampled any time at any section of the “gut” without ethical constraints. On the other hand, it is acknowledged that since microbial changes can be affected by different factors in the host and the system lacks many gut components, up to 80% of bacteria species may be difficult to be cultured in vitro, therefore HCM cannot provide a perfect simulation, but is a relatively robust option to study the interaction between human gut microbiota and xenobiotics (such as the dietary components we tested here). We also fully acknowledge that additional validation studies in animal models and clinical trials should be performed before more practical recommendations can be provided for health care purposes.

### 3.2. Tracking the Phenolic Compounds Contents in Gut Microbiota

**Figure 2:**
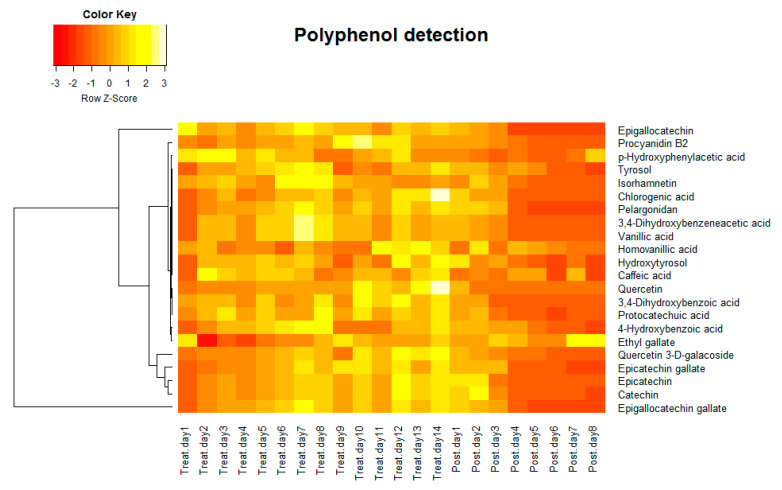
Heat map of average relative polyphenol density of three vessels detected by HPLC-MS/MS.

Samples collected from the treatment period and post-treatment period were analyzed by a targeted HPLC-MS/MS polyphenol method to detect relative polyphenol concentration. The targeted analysis identified 22 polyphenols that were differentially expressed in the HCM in the treatment period and post-treatment period. As is shown in the heat map (Figure 2), the relative concentration of polyphenols in the HCM (the average of all three vessels) increased through treatment days and decreased post-treatment. Polyphenols have attracted great interest from researchers due to their antioxidant properties [59]. In addition to their antioxidant properties, others also showed that they can modulate the activity of a wide range of enzymes and cell receptors [60], and this motivated researchers to study the health effects of polyphenol consumption. It is important to realize that the high abundance of polyphenols in the diet does not necessarily guarantee their bioavailability and bioactivities in the human body. This is mostly because of either the lower efficiency of absorption by the human body or their low intrinsic activity [61]. Typically, most polyphenols cannot be absorbed in their native forms, which present as esters, glycosides, or polymers. Intestinal enzymes or colonic microflora can hydrolyze these substrates and help them be absorbed [61]. Our results indicate that the gut microflora took up polyphenols during the polyphenol treatment period, and maintained relatively stable intracellular polyphenol concentrations. The intracellular polyphenol abundance dropped once the treatment was stopped.

### 3.3. Microbial Composition Changes in Response to Green Tea Polyphenols

**Figure 3:**
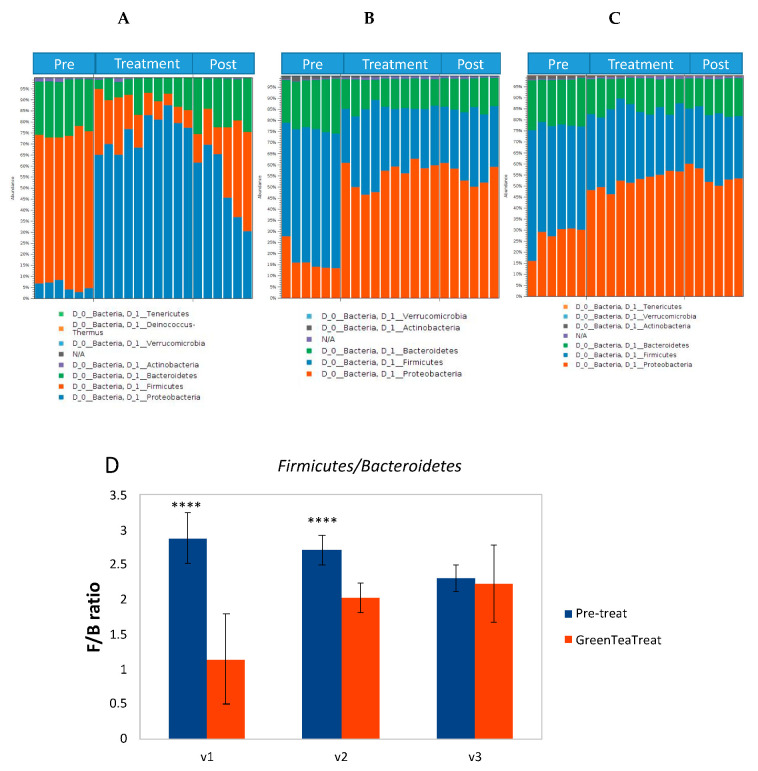
Bar charts representing bacterial taxonomy based on the 16 S rRNA gene at the phylum level in 3 parts of HCM ((**A**). Vessel 1; (**B**). Vessel 2; (**C**). Vessel 3). (**D**). *Firmicutes/Bacteroidetes* ratio, *n* = 6 for pretreatment samples, and *n* = 10 for GTE treatment samples. DNA was isolated from vessel contents for MiSeq sequencing. The relative abundance of bacteria was calculated based on the operational taxonomic unite (OTUs) (**** *p* < 0.001).

Bacterial DNA in samples collected from HCM was isolated for Illumina Miseq sequencing. The relative abundance of bacteria in different taxonomic levels was calculated based on the operational taxonomic unit (OTUs) to investigate the effect of green tea treatment on gut microbiota. After several steps of data processing, a bacterial taxonomic overview of gut microbial composition based on the 16 S rRNA gene at the phylum level in three parts of the HCM (A. Vessel 1; B. Vessel 2; C. Vessel 3) is represented by bar charts in Figure 3. The phyla in our study were mainly identified as *Proteobacteria*, *Firmicutes*, *Bacteroidetes*, *Actinobacteria*, and *Verrucomicrobia* from these HCM samples, of which *Actinobacteria*, *Bacteroidetes*, *Firmicutes*, and *Proteobacteria* are known to comprise approximately 93.5% of human gut microbiota [62]. The exposure of green tea polyphenols to gut microbiota resulted in a significant decrease in the abundance of *Firmicutes* and a slight decrease in the abundance of *Bacteroidetes*. In a previous report, a lower relative abundance of the *Bacteroidetes* in obese subjects and the increased relative abundance of *Bacteroidetes* were reported to change progressively, and proportionally to the degree of weight loss [63]. *Firmicutes* are mainly Gram-positive bacteria, so these observations showed that green tea polyphenols may be more inhibitory to Gram-positive bacteria. Moreover, the significant decrease in the *Firmicutes/Bacteroidetes* ratio in vessel 1 and vessel 2, and a non-significant slight decrease in vessel 3, was observed from pretreatment groups compared to green tea polyphenols-treated groups (Figure 3D). Some studies have reported that the *Firmicutes/Bacteroidetes* ratio was positively correlated with the obese phenotype independently of diet [64]. This result indicates that a possibly beneficial shift in gut microbial communities was obtained after the green tea polyphenol treatment.

**Figure 4:**
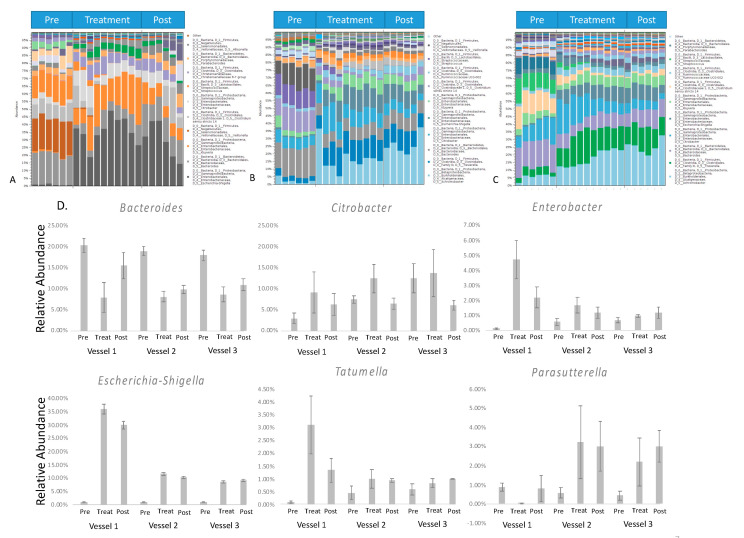
Bar charts representing bacterial taxonomy based on the 16 S rRNA gene at the genus level in 3 parts of the HCM ((**A**). Vessel 1; (**B**). Vessel 2; (**C**). Vessel 3). (**D**). The relative abundance of the significant genus in a three-time period. DNA was isolated from vessel contents for MiSeq sequencing. The relative abundance of bacteria was calculated based on the operational taxonomic unit (OTUs). The *Y*-axis represents the relative abundance of each detected genus.

Taxonomic assignment identified more than 20 genera that presented in all of the samples among OTUs. Among all sequences, 90% were mapped against OTUs with the assigned genus. The top 10 most dominant genera are shown in the summary figure (Figure 4A–C). The detailed vessel to vessel changes of a few dominant bacteria genera, such as *Escherichia-Shigella, Citrobacter, Enterobacter, Tatumella,* and *Parasutterella*, is presented in Figure 4D to show the changes in the relative abundance of six genera after green tea polyphenols treatment.

**Figure 5:**
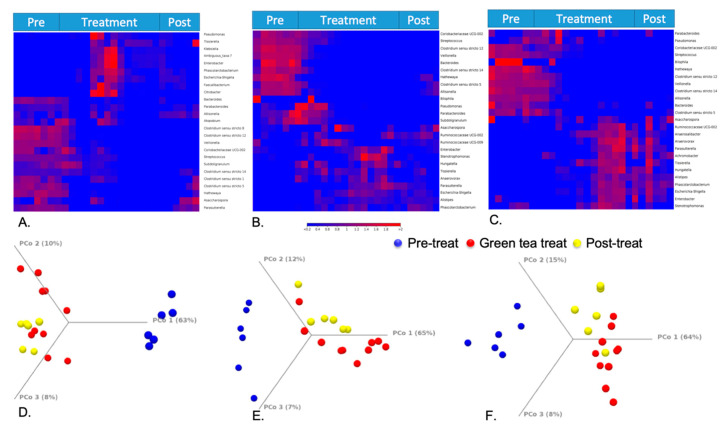
Heat map of taxonomical assignments of 3 parts of the HCM ((**A**). Vessel 1; (**B**). Vessel 2; (**C**). Vessel 3). Beta diversity results by principal coordinate analysis ((**D**). Vessel 1; (**E**). Vessel 2; (**F**). Vessel 3).

The hierarchical clustering is based on Euclidean distance and complete linkage to present the modulation of these bacterial expression profiles under different treatment periods in HCM vessels 1–3, respectively (Figure 5A–C). In the heat map, each row corresponds to a feature and each column to an HCM sample. The colors reflect data information in a two-dimensional matrix, which can visually represent the size of data values with a defined color scale. Overall, the result shows that the relative abundances of most genera among three vessels have changed through the treatment days. Beta diversity was analyzed using a Bray–Curtis metric and showed clear differences among samples from different treatment periods. Furthermore, the principal coordinate analysis (PCoA) in Figure 5D–F enables us to visualize the effects of green tea polyphenols by showing that pretreatment samples tend to form a cluster separated from green tea polyphenol-treated samples and post-treatment samples in three vessels. The observations of green tea-induced gut microbiome changes in this study can be supported by both animal studies and clinical trials from other publications [65,66], and can potentially provide an underlying mechanism to understand how green tea ingestion could favorably regulate the profile of the gut microbiome and help to offset dysbiosis triggered by obesity or high-fat diets. Similar to previous studies [66,67], our results indicated that the gut microbial composition changes induced by short-term GTE treatment may increase the *Firmicutes/Bacteroidetes* ratio that is positively correlated to the metabolic health of the host, and significantly reduces the markers of inflammation and bacterial translocation [67]. During and after the GTE treatment, the increased abundance of short-chain fatty acid (SCFA)-producing bacteria, such as *Ruminococcaceae*, in our results may also suggest that GTE can benefit the host’s gut health by facilitating the increased production of SCFAs that can be utilized by enterocytes as energy sources in the colon [68].

### 3.4. Green Tea Polyphenol-Induced Gut Microbial Metabolic Changes and Their Association with Microbial Composition Changes

**Figure 6:**
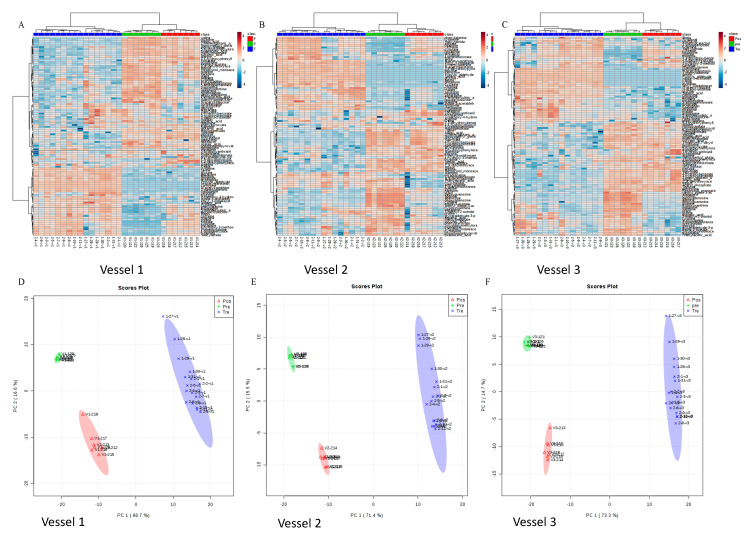
Heat map presentation of intracellular metabolic profiles computed by Euclidean distance and Ward’s hierarchical agglomerative clustering method (**A**. Vessel 1; **B**. Vessel 2; **C**. Vessel 3). Principal component analysis (PCA) of 130 targeted metabolites comparison for pretreatment days, green tea treatment days, and post-treatment days (**D**. Vessel 1; **E**. Vessel 2; **F**. Vessel 3).

Out of 230 targeted intracellular metabolites, 173 were detected from all the samples (Supplementary Table S1). The metabolites in more than 75% of samples were then used for statistical analyses. In Figure 6, an overview of the metabolic profile from each sample in each vessel is show in the heat map. Each column represents one sample and each row represents one targeted metabolite. The comparison of three groups in three vessels indicates dramatic changes in the metabolic activities of the studied gut microbes post green tea treatment. Differences in the metabolic profiles from both pretreatment samples and post-treatment samples compared to green tea-treated samples were observed. Principle component analysis (PCA) was performed to confirm that the metabolic activities were affected by exposure to green tea polyphenols (Figure 6). Six representative metabolites that have statistical significance in three experimental phases were shown in Supplementary Figure S1. These results showed that changes in the microbial metabolic profiles of polar metabolites after being fed green tea polyphenols were most pronounced during the 10-day continuous feeding period. Some of these detected metabolites were also reported previously. For example, 3-methyladenine, one of the top 10 metabolites which was significantly increased in the treatment period, showed a significant difference between pre- and post-treatment period, and is a PI3-kinase inhibitor that inhibited the formation of LC-3 II, which can be stimulated by EGCG [69]. Xanthine is an extremely weak basic (essentially neutral) compound (based on its pKa), which exists in all living species, ranging from bacteria to humans. Xanthine has been found to be associated with several diseases, such as xanthinuria type II disease, hydrocephalus and eosinophilic esophagitis in humans; it has also been linked to inborn metabolic disorder. The reduction in this metabolite during the GTE intervention indicated that potential health benefits may be achieved by green tea intake and its consequential regulation of microbial xanthine production. Meanwhile, urocanate, an intermediate of the histidine degradation pathway, has been reported to serve as a molecule that promotes bacterial infection via molecular interaction with the bacterial regulatory protein HutC [70]. It has also been suggested to be central to the elicitation of bacterial pathogenesis, in addition to being a valuable source of carbon and nitrogen. The generally decreasing trends of uracanate in our study could therefore be considered as potentially beneficial changes to human health that are induced by gut microbial-dependent GTE intervention.

The significantly changed metabolites measured, and the relative microbial genus abundances, were used to compute a spearman rank-based correlation matrix. There are several associations between microbial population and metabolite activity that can be observed in Supplementary Figure S2. Spearman’s analyses revealed that the abundances of *Enterobacter*, *Citrobacter* and *Tatumella* were inversely correlated with adipic acid, urocanate and xanthine, respectively. A recent study showed that urocanate, in conjunction with protein HutC, can play a critical role in the global control of cell motility, cellular metabolism, and the expression of virulence factors within the zoonotic pathogen *Brucella abortus* and opportunistic human pathogen *Pseudomonas aeruginosa* [70]. Our results for urocanate may suggest that *Citrobacter* can inhibit opportunistic pathogens and reduce the production of urocanate, and consequentially modulate the signaling pathways that the eukaryotic hosts utilized for their bacterial recognitions [70]. Meanwhile, the abundances of *Escherichia-Shigella, Parasutterella* and *Bacteroides* were positively correlated with thiamine, sorbate, and 2′, 4′-dihydroxy acetophenone. The positive correlation of *Escherichia-Shigella* and thiamine (vitamin B1) production during GTE intervention (both have increased levels) may suggest the effect of GTE on promoting *Escherichia-Shigella* in biotransforming diets and foods into vitamin B1, which possesses antioxidant and detoxification activities [71], and which consequentially could benefit the health of the host. These findings can be used to set up new hypotheses to be tested in future studies that connect microbial population/diversity to the microbial metabolic products.

### 3.5. Functional Prediction of the Gut Microbiota

**Figure 7:**
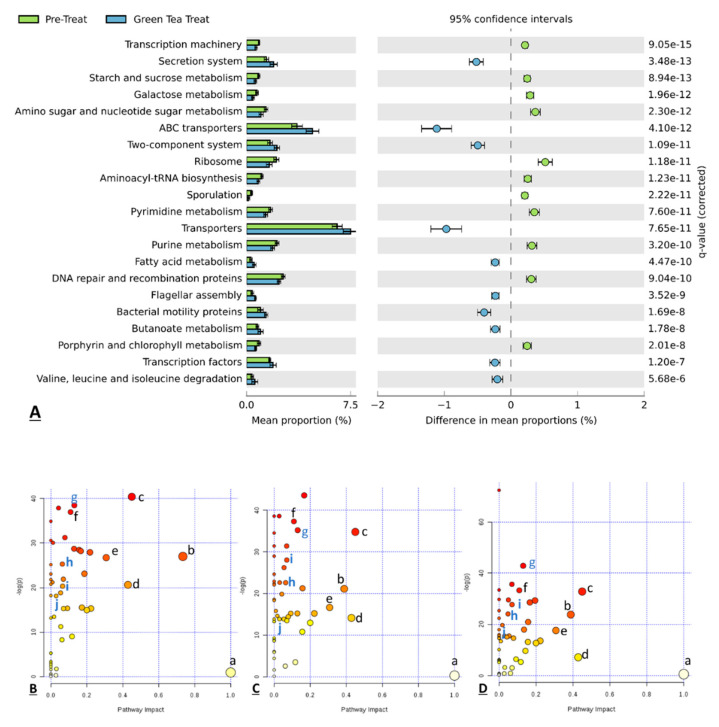
The difference in the mean proportion of microbial functions between the pretreatment samples and treated samples along with the associated 95% confidence interval of this effect size, *q*-value below 0.005, and the difference between proportions above 0.2 (**A**). Representative pathway analysis indicated major pathways in the comparison of pretreatment samples vs. green tea treated samples ((**B**–**D**) for vessel 1, vessel 2, and vessel 3). Some common pathways of three vessels that have –log(*p*) above 10 are labeled in lower case letters as: *a.* Inositol phosphate metabolism; *b.* Alanine, aspartate, and glutamate metabolism; *c.* Glycine, serine, and threonine metabolism; *d*. Biotin metabolism; *e*. beta-Alanine metabolism; *f.* Cysteine and methionine metabolism; *g.* Aminoacyl-tRNA biosynthesis; *h.* Pyrimidine metabolism; *i.* Purine metabolism; *j.* Butanoate metabolism.

To further analyze the functional profiling of microbial communities, the functional composition of the metagenome was predicted by the computational approach PICRUST (phylogenetic investigation of communities by reconstruction of unobserved states) by using marker gene data and databases [53]. Gene-functional data were input into the STAMP software package. Figure 7A shows the featured pathways that were significantly different between the pretreatment samples and green tea-treated samples. Welch’s *t*-test, a *q*-value filter, and an effect size filter were used for statistical analysis. After the features with *t*-test *p*-values > 0.05, *q*-values > 0.005 and differences between proportions <0.2 were removed, some remarkable biological functions related to green tea exposure can be obtained from the result. Twenty-one functional pathways were found as expressed significantly different between two groups. Comparing predicted metagenomes, the microbiota of samples collected from the treatment period were predicted to have a great capability for secretion system, ABC transporters, transporters, two-component system, fatty acid metabolism, flagellar assembly, bacterial motility proteins, butanoate metabolism and transcription factors, compared to samples collected from the pretreatment period. On the other hand, the microbiota of samples collected from the pretreatment period were predicted to have a great capability for transcription machinery, starch and sucrose metabolism, galactose metabolism, amino sugar, nucleotide sugar metabolism, ribosome, Aminoacyl-tRNA biosynthesis, Sporulation, Pyrimidine metabolism, purine metabolism, Peptidases, DNA repair, recombination proteins and porphyrin and chlorophyll metabolism, compared to samples collected from the pretreatment period. These results illustrated that the microbial composition change induced by green tea polyphenol also changes the functional roles of these microorganisms. Besides, we also performed an analysis of metabolic pathways making connections among metabolites based on detected metabolites by using MetaboAnalyst 3.0. Figure 7B–D show 10 common pathways that have a –log(*p*) above 10 shared by all three vessels. Among these pathways, aminoacyl-tRNA biosynthesis, pyrimidine metabolism, purine metabolism and butanoate metabolism were also functional pathways predicted by Picrust. In our study, several significantly regulated metabolic pathways were amino acids (AAs) metabolic pathways. AAs can act as precursors for many metabolic end products in biochemical reactions involving the host intestinal mucosa and gut microbiota [72]. After passing through the epithelial barrier, microbial AA metabolites can enter and accumulate in the host circulation, where they can be sensed by host immune cells and then induce a variety of biological functions via different receptors and mechanisms. Alternations in AA composition and abundance can affect AA-metabolizing bacterial communities, as well as modulate macrophages and dendritic cells via the autoinducer-2 (AI-2), toll-like receptors (TLRs) and NOD-like receptors (NLRs) of the host [73], and also regulate the gut–microbiome–immune axis via serotonin/5-hydroxytryptamine (5-HT), aryl hydrocarbon receptor (AhR), and other signaling pathways [72], all of which may play critical roles, directly or indirectly, in regulating the gut microbiota and the host intestinal mucosal immunity, and modulating the intestinal homeostasis of the host.

## 4. Conclusions

In this study, we demonstrated the utilization of a human colonic model (HCM) system for the investigation of interactions between dietary components and human gut microbes. The pH, temperature, anaerobic environment, and the volume/timing of feeding in each compartment of the system were tightly controlled to allow a study of the impact of green tea polyphenols on the gut’s microbial population and its metabolites. In addition, the polyphenols and their microbial metabolic products from gut microbial fermentation were also detected and reported. As the gut’s microbial composition and its metabolic profile showed significant responses to the green tea polyphenol treatment, the result also illustrated that the changes in microbial composition induced by green tea polyphenol can also change the functional roles of these microorganisms. Overall, we believe this system can be an excellent tool for in vitro microbial metabolism analysis and can contribute significantly to future food and nutritional research, as demonstrated in this green tea study. Through the implementation of this HCM system, the human gut microbiota can be studied dynamically and continuously within a fully controlled environment. However, the system still has some limitations to perfectly mimicking the real situation in the gut. The use of the HCM system would help to minimize, and now fully replace, animal testing, but certain parameters would still have to be studied with in vivo animal models to understand the roles the host may play in these complex interactions.

## Supplementary Materials

The following are available online, Figure S1: The relative abundance of significant metabolites (ANOVA *p*-value < 1 × 10^−10^) that correlate with the genus in three vessels of HCM, Figure S2: Correlation analysis demonstrating the relationships between the significantly altered gut microbes and the detected microbial metabolites from the HCM study, Table S1: A list of one hundred and seventy-three detected metabolites reported in this study, Table S2. A list of seventy-seven metabolites form frequently investigated metabolic pathways.

## Sample Availability

Samples of the compounds used for targeted analysis are available from the commercial vendors or from the authors upon reasonable request.
