# Supplementary material for: Monitoring the Diversity and Metabolic Shift of Gut Microbes during Green Tea Feeding in an In Vitro Human Colonic Model
Source: Molecules. 2020 Nov 3;25(21):5101. doi: 10.3390/molecules25215101 (PMC7663002; doi:10.3390/molecules25215101)
Supplement: Supplementary file 1 [file molecules-25-05101-s001.pdf]

Supporting information for

Monitoring the Diversity and Metabolic Shift of Gut Microbes during Green Tea Feeding in an *in vitro* Human Colonic Model

Mengyang Xu[1]#, Kundi Yang[1]#, and Jiangjiang Zhu [2,3] *

1. Department of Chemistry and Biochemistry, Miami University, Oxford, OH, 45056

2. Human Nutrition Program, Department of Human Sciences, The Ohio State University, Columbus, OH, 43210

3. James Comprehensive Cancer Center, The Ohio State University, Columbus, OH, 43210

# These two authors contributed equally to this work

* Corresponding author, Email: zhu.2484@osu.edu; Tel: 614-685-2226

Legends of Supplementary Figures:

Figure S1. The relative abundance of significant metabolites (ANOVA P-value <1E10) that correlate with the genus in three vessels of HCM.

Figure S2. Correlation analysis demonstrating the relationships between the significantly altered gut microbes and the detected microbial metabolites from the HCM study.

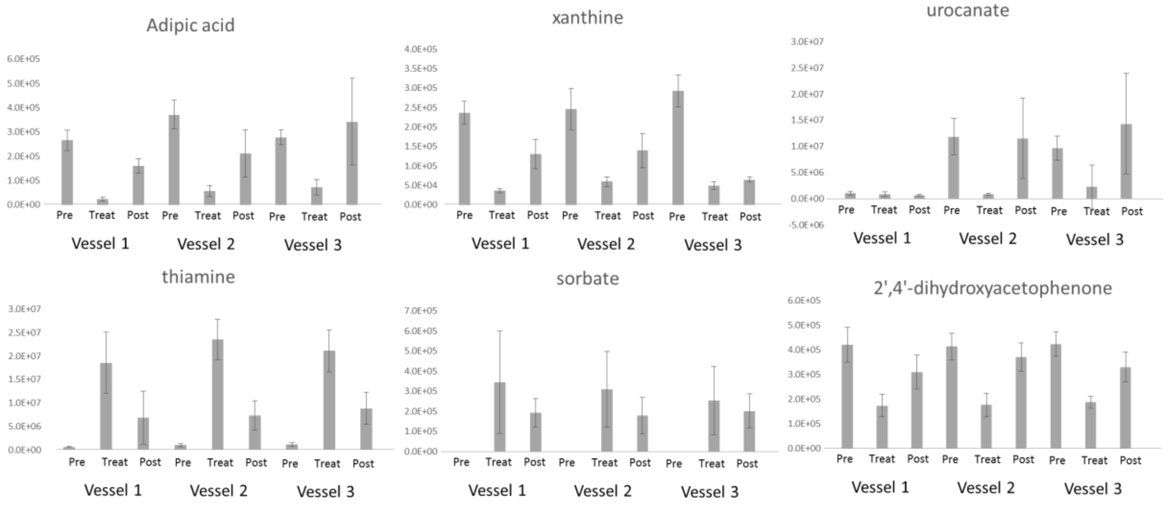
Adipic acid
xanthine
urocanate
thiamine
sorbate
2',4'-dihydroxyacetophenone
Pre
Treat
Post
Vessel 1
Vessel 2
Vessel 3

Figure S1

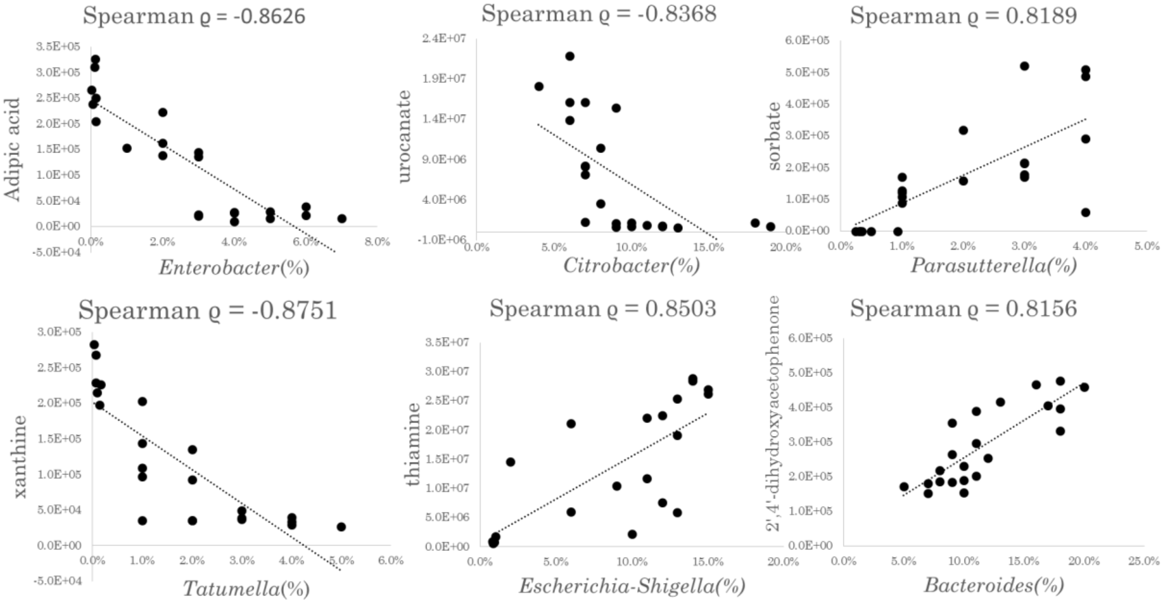

Spearman ϱ = -0.8626
Adipic acid
Enterobacter(%)
Spearman ϱ = -0.8368
urocanate
Citrobacter(%)
Spearman ϱ = 0.8189
sorbate
Parasutterella(%)
Spearman ϱ = -0.8751
xanthine
Tatumella(%)
Spearman ϱ = 0.8503
thiamine
Escherichia-Shigella(%)
Spearman ϱ = 0.8156
2',4'-dihydroxyacetophenone
Bacteroides(%)

Figure S2

Table S1. A list of one hundred and seventy-three detected metabolites reported in this study

#	Metabolite
1	lipoamide
2	2-aminoethyl phosphonate
3	S-nicotine
4	phosphoenolpyruvate
5	caffeine
6	phenylpyruvate
7	thymine
8	4-hydroxybenzoate
9	deoxyadenosine
10	oxaloacetic acid
11	deoxyguanosine
12	methylmalonate
13	succinic acid
14	urocanate
15	lauroylcarnitine
16	nicotinamide
17	tyramine
18	pyruvate
19	5-hydroxymethyluracil
20	riboflavin
21	2,3-dihydroxybenzoic acid
22	trigonelline
23	adenine
24	adenosine
25	hypoxyxanthine
26	4-aminobenzoate
27	alpha-ketoglutaric acid
28	dopamine
29	cytidine
30	noradrenaline
31	pterin
32	creatinine
33	guanosine
34	N-acetyl-glucosamine
35	pyridoxine
36	xanthine
37	purine
38	5-aminolevulinic acid
39	cytosine
40	uracil

41	histamine
42	indole-3-acetate
43	sorbate
44	Maleic acid
45	cyclic AMP
46	dAMP
47	guanine
48	uridine
49	3-dehydroshikimate
50	dihydroorotate
51	cyclic CMP
52	malate
53	homocysteine
54	fumarate
55	cyclic GMP
56	guanidinoacetate
57	tryptamine
58	dGMP
59	myo-inositol
60	5-aminopentanoate
61	thiamine
62	glucose
63	carnosine
64	acetylcholine
65	cysteine
66	4-guanidinobutanoate
67	ethanolamine
68	pentanoate
69	glucosamine-6-phosphate
70	serotonin
71	citrulline
72	glyceraldehyde-3-phosphate
73	glutathione
74	FAD
75	melatonin
76	3-hydroxyanthranilate
77	octopamine
78	CYS-GLY
79	acetyl-CoA
80	AMP
81	theophylline
82	dCMP
83	GMP

84	allantoin
85	ADP
86	3-phosphoglyceric acid
87	F6P
88	NAD
89	UDP-N-acetylglucosamine
90	CMP
91	G6P
92	dGDP
93	UDP-glucose
94	stachyose
95	N-acetyl-ornithine
96	3-ureidopropionate
97	ornithine
98	4-aminobutanoate
99	cadaverine
100	S-adenosyl-methionine
101	putrescine
102	NADPH
103	glutathione disulfide
104	1-methyl-6,7-dihydroxy-1,2,3,4-tetrahydroisoqui
105	2',4'-dihydroxyacetophenone
106	2,6-dihydroxypyridine
107	2-methylglutaric acid
108	2-methylmaleate/itaconate
109	2-oxoadipate
110	3-(4-hydroxyphenyl)lactate
111	3-alpha,11-beta,17-alpha,21-tetrahydroxy- 5-al
112	3-hydroxybenzoate
113	3-hydroxyphenylacetate
114	3-methoxy-4-hydroxymandelate
115	3-methyl-2-oxindole
116	3-methyl-2-oxovaleric acid
117	3-methyladenine
118	3-methylbutanal
119	4-hydroxy-3-methoxyphenylglycol
120	4-methyl-2-oxovaleric acid
121	4-methylcatechol
122	4-quinolinecarboxylic acid
123	5-hydroxyindoleacetate
124	Acetyl phosphate
125	Adipic acid
126	alanine

127	arginine
128	asparagine
129	aspartic acid
130	azelaic acid
131	biotin
132	cinnamaldehyde
133	cysteine
134	dethiobiotin
135	D-gulonic acid gama-lactone
136	Diacetyl
137	ethyl 3-indoleacetate
138	glutamic acid
139	glutamine
140	glycine
141	histidine
142	homogentisate
143	homovanillate
144	indole-3-acetaldehyde
145	leucine/isolucine
146	lysine
147	mannitol
148	mannose
149	methionine
150	methyl indole-3-acetate
151	mono-methyl glutarate
152	myo-inositol
153	N,N-dimethyl-1,4-phenylenediamine
154	N-acetyl-l-leucine
155	N-acetylserotonin
156	phenethylamine
157	phenylacetic acid
158	phenylalanine
159	proline
160	psicose
161	Pyridoxal
162	pyruvic aldehyde
163	resorcinol monoacetate
164	ribitol
165	salicylamide
166	salicylate
167	serine
168	sorbose
169	suberic acid

170	threonine
171	tryptophan
172	tyrosine
173	valine

Table S2. A list of seventy-seven metabolites form frequently investigated metabolic pathways

#	Metabolite
1	lipoamide
2	phosphoenolpyruvate
3	caffeine
4	phenylpyruvate
5	4-hydroxybenzoate
6	deoxyadenosine
7	oxaloacetic acid
8	methylmalonate
9	succinic acid
10	urocanate
11	tyramine
12	5-hydroxymethyluracil
13	riboflavin
14	2,3-dihydroxybenzoic acid
15	trigonelline
16	adenine
17	adenosine
18	hypoxyxanthine
19	4-aminobenzoate
20	dopamine
21	noradrenaline
22	pyridoxine
23	xanthine
24	5-aminolevulinic acid
25	cytosine
26	uracil
27	histamine
28	sorbate
29	indole-3-acetate
30	guanine
31	3-dehydroshikimate
32	malate
33	myo-inositol
34	thiamine
35	glucose
36	acetylcholine
37	serotonin
38	glyceraldehyde-3-phosphate
39	3-hydroxyanthranilate
40	octopamine
41	2',4'-dihydroxyacetophenone

42	2-methylglutaric acid
43	3-(4-hydroxyphenyl)lactate
44	3-hydroxybenzoate
45	3-hydroxyphenylacetate
46	3-methyl-2-oxindole
47	4-hydroxy-3-methoxyphenylglycol
48	5-hydroxyindoleacetate
49	Adipic acid
50	alanine
51	arginine
52	asparagine
53	aspartic acid
54	Azelaic acid
55	Biotin
56	dethiobiotin
57	glutamine
58	Homogentisate
59	Homovanillate
60	leucine/isolucine
61	Mannitol
62	Mannose
63	methionine
64	Methyl indole-3-acetate
65	Myo-inositol
66	Phenylacetic acid
67	phenylalanine
68	proline
69	psicose
70	Pyridoxal
71	Resorcinol monoacetate
72	Ribitol
73	Salicylamide
74	Salicylate
75	threonine
76	tryptophan
77	tyrosine
